# Legal knowledge, needs, and assistance seeking among HIV positive and negative women in Umlazi, South Africa

**DOI:** 10.1186/s12914-016-0077-z

**Published:** 2016-01-22

**Authors:** Lauren M. Hill, Suzanne Maman, David Holness, Dhayendre Moodley

**Affiliations:** Department of Health Behavior, Gillings School of Global Public Health, University of North Carolina at Chapel Hill, 331 Rosenau Hall, CB #7440, Chapel Hill, 27599 NC USA; University of KwaZulu-Natal Law Clinic, Durban, South Africa; Department of Obstetrics and Gynaecology, Nelson R. Mandela School of Medicine, University of KwaZulu-Natal, Durban, South Africa

**Keywords:** South Africa, Women, HIV, Legal knowledge, Legal rights

## Abstract

**Background:**

The rights of women and people living with HIV (PLHIV) are protected under South African law, yet there is a gap in the application of these laws. While there are numerous systemic and social barriers to women's and PLHIV's exercise of their legal rights and rights to access social services, there has been little effort to document these barriers as well as legal needs and knowledge in this context.

**Methods:**

1480 HIV-positive and HIV-negative women recruited from an antenatal clinic in Umlazi Township completed a questionnaire on legal knowledge, experience of legal issues, assistance seeking for legal issues, and barriers to seeking assistance. We compared the legal knowledge and experience of legal issues of HIV-positive and HIV-negative women, and described assistance seeking and barriers to assistance seeking among all women.

**Results:**

Both HIV-positive and HIV-negative women had high levels of knowledge of their legal rights. There were few important differences in legal knowledge and experience of legal issues by HIV status. The most common legal issues women experienced were difficulty obtaining employment (11 %) and identification documents (7 %). A minority of women who had ever experienced a legal issue had sought assistance for this issue (38 %), and half (50 %) of assistance sought was from informal sources such as family and friends. Women cited lack of time and government bureaucracy as the major barriers to seeking assistance.

**Conclusions:**

These results indicate few differences in legal knowledge and needs between HIV-positive and HIV-negative women in this context, but rather legal needs common among women of reproductive age. Legal knowledge may be a less important barrier to seeking assistance for legal issues than time, convenience, and cost. Expanding the power of customary courts to address routine legal issues, encouragement of pro bono legal assistance, and introduction of legal navigators could help to address these barriers.

## Background

South Africa’s legal environment concerning both the rights of women and the rights of people living with HIV/AIDS (PLHIV) has been cited as a potential model for other countries on the continent [1]. Despite favorable legislation there are significant barriers to the application of these laws [1], and PLHIV continue to be subject to unfair treatment in South Africa. Such discrimination is in direct contradiction to the right to equality provided for in South Africa’s constitution [2, 3], and is found on multiple fronts, at the workplace, in healthcare, and in school [4]. Unfair termination and refusal of employment and health care services on the basis of HIV-status has been documented in South Africa [4], as has discrimination in schools often in the form of enrollment denial or differential treatment by instructors [4]. Such concerns affect a significant proportion of women of reproductive age living in South Africa as it is estimated that 1 out of every 3 young women in the country is living with HIV [5]. Women between the ages of 25–29 years are the most vulnerable to HIV acquisition with national HIV prevalence estimates for this age group ranging from 32 to 34 % [5]. Forty-two percent of women seeking antenatal care services at Umlazi Section D clinic in KwaZulu-Natal, an urban primary health care clinic in South Africa, are living with HIV.

Prior to 1994 and the advent of democracy in South Africa, there were few laws protecting the rights of PLHIV in South Africa, but since then legislation related to the rights of PLHIV has made gradual but substantial progress with the introduction of multiple pieces of legislation aimed to protect PLHIV’s rights to employment, education, social services, and health care [6–9]. In spite of the proliferation of laws protecting PLHIV over the past two decades, the majority of those living with HIV or most at risk for HIV often do not have the knowledge that such legal protection exists [10]. Further, PLHIV in South Africa are often subject to multiple social vulnerabilities, as people with lower income and socioeconomic status are more likely to be HIV-positive than their wealthier counterparts [11]. These social disadvantages may also make PLHIV more legally vulnerable than the general population for reasons beyond their HIV status. Experts suggest that for legal reform targeting HIV-related discrimination and human rights to have the greatest impact, it must be accompanied by public education [10]. The South African National HIV/AIDS Strategic Plan for 2012–2016 similarly identified the need for greater rights awareness in 4 key areas: employment, education, health care, and social services [12, 13]. Such calls have been made in the past by the same body but evaluations of past awareness campaigns suggest that these attempts have fallen short of their goals [12, 13], calling for additional attempts to raise rights awareness among PLHIV [13].

In addition to highlighting the need to promote the rights of PLHIV, the South African National AIDS Council has also acknowledged the legal vulnerabilities of all South African women which relate to their heightened risk for HIV infection, particularly pointing to legal issues related to intimate partner violence (IPV - acts of physical, psychological, and sexual abuse by sexual partners [14]) and gender inequality [13]. There are laws protecting women from discrimination on the basis of gender [9] and against rape and other types of IPV [15]. The difficulty that South African women face in exercising their rights seems to rest not in a lack of laws protecting them but rather in barriers to the application of these laws [16]. Despite the existence of gender equity and other laws protecting women’s rights, women remain a highly vulnerable population in South Africa and limits to the ability to create *de facto* gender equity in South Africa have limited women’s ability to exercise their legal rights [17]. Calls have also been made for further education of women with respect to their legal rights relevant to their sexual health [18].

There are reasons why South African women may be unaware of their rights or be unable to exercise them. Legal jargon can be complicated and difficult to understand even for those who are literate [1]. This is compounded by the fact that South Africa has eleven official languages and yet laws are not always translated into all eleven official languages in South Africa, making comprehension difficult for those not fluent in the languages in which a law is published. Furthermore, even those individuals who understand their legal rights often face barriers to enforcing them. Even the most routine legal procedures can be complicated and require extensive documentation, such as birth certificates and government identification documents (IDs), which not all women may have and may be difficult and costly to access. Additionally, private legal counsel, while widely available in South Africa, is largely unaffordable to the majority of the population. Legal aid is provided by the government, but primarily for criminal rather than civil cases; as women are rarely involved in criminal cases, this aid largely does not serve the legal needs of women [19]. Further, the delivery of legal aid has been noted to be plagued by inefficiencies and bureaucracy [20]. In short, the legal system is largely unavailable to many women in South Africa, and women are under-informed about their rights [21].

Though gaps in accessibility of the legal system are widely acknowledged in South Africa, little is known about legal knowledge, variation in legal needs by HIV-status, experiences of legal issues, and assistance seeking for legal issues among women in this context. We sought to understand the legal issues faced by HIV-positive and HIV-negative women of reproductive age and to assess their legal knowledge, needs, and experience of discrimination through a questionnaire with pregnant and postpartum women in one antenatal clinic in Umlazi township, South Africa. Our findings have implications for future programs attempting to address the legal needs of this population and in similar resource-limited settings.

## Methods

### Background and setting

This research was conducted in the context of a parent study, the South Africa HIV/AIDS Antenatal Post-test Support study (NIH R01 HD050134). The study took place in Umlazi Township, Durban, which is the second largest township in South Africa [22], with an estimated population reaching nearly 2,000,000 [22]. In the township pregnant women receive antenatal care at one of 17 primary health care clinics in the township, are referred to the hospital for delivery, and return to the clinics for postpartum care. Women in the study were recruited from a public antenatal clinic in the township. The data presented here were collected within a 5 year intervention trial designed to evaluate the efficacy of enhanced post-test support for HIV-positive and HIV negative pregnant and postpartum women. A component of the post-test support provided in the trial was legal information and legal counsel if required. Through a partnership with the University of KwaZulu-Natal Law Clinic, a lawyer was available at the clinic to meet with women in the intervention arm. The intervention was evaluated as a package of services, which included psychosocial counseling in addition to the legal assistance and counsel. Primary outcome results for the trial have been published elsewhere [23]. Women’s knowledge of their legal rights and their legal needs were assessed among all 1480 women enrolled in the trial at baseline. The purpose of this paper is to describe and compare HIV-positive and HIV-negative women’s legal knowledge and legal needs. Through this analysis we aimed to gain a better understanding of women's knowledge of their legal rights, knowledge of legal recourse, perceived legal need, and experiences of discrimination.

### Sample

The data set for this analysis comes from the baseline assessment of SAHAPS. Recruitment for the trial began in May 2008 and ended in June 2010. Women were recruited at their first antenatal visit, which on average took place when women were 5 1/2 months pregnant. Inclusion criteria to participate were: 1) at least 18 years old; 2) had never tested for HIV or had tested negative for HIV at least 3 months prior to recruitment; 3) attending first antenatal visit when HIV testing was offered; 4) had a primary partner who they had been with for at least 6 months; 5) planned to live in Durban for at least the next year; 6) planned to bring their infant to the clinic for immunizations; 7) were able to communicate in isiZulu or English; and 8) were not a high-risk pregnancy. Women participating in the study completed a baseline assessment immediately after providing informed consent. In total 1480 women were included in the trial.

### Measures

Based on participant preference, the assessment was conducted in isiZulu or English by one of four trained South African survey interviewers using computer assisted personal interviews (CAPI). Each interviewer completed training on conducting quantitative interviews, which included sessions on rapport building, asking sensitive questions, and good ethical conduct. Women who showed signs of distress during the interview were referred to an onsite psychologist. At baseline and at 14-weeks postpartum we measured knowledge of legal rights, knowledge of legal recourse, perceived legal need, and experiences seeking assistance for legal issues (formal and informal help with legal problems encountered). For the descriptive purposes of this paper, only baseline data are presented here. Questionnaire items were measured through the series of questions included in Table 1.

**Table 1:** Legal questionnaire items

*Domain*	*Questions*
Legal needs/ assistance seeking	• Have you ever had a problem with (check all that apply): housing, work discrimination, getting work, getting a residence permit, school discrimination, abusive partner, divorce, maintenance, custody, making a will, healthcare discrimination, obtaining an ID? • If yes, have you received assistance for this problem? If yes, from whom? If not, why not?
Legal knowledge	• Do you think it is against the law for a man to beat his partner? • If a woman is being beaten by her partner, can (response category) help the woman to stop the beating? • If a man and a woman have a child together, is it against the law if the man does not give the woman any money or support for the child? • Is there anything the woman can do to make the man provide child support? What can she do? • If a woman’s husband passes away, does the law allow her to receive his death benefits? • If a man and a woman get divorced, is there a law that protects the woman if she wants to keep the child? • If the male partner wants to take the children away but the female does not consent, how can the female stop him from taking the children? • Does the law have a say in what happens to people’s possessions after they pass away? • How can a person use the legal system to help make sure that their possessions go to right people after they pass away?

Legal issues addressed in the questionnaire were selected through formative interviews that we conducted with key informants at legal aid organizations in Durban prior to implementing this study. The domains included represent the most common legal issues these organizations reported helping women address through their services. Some of these issues are not strictly legal as they would not necessarily require a court case to resolve, as in the case of difficulty obtaining an ID document or difficulty obtaining housing. Rather, many of these issues relate to barriers to accessing social services and exercising personal rights for which women may need access to lawyers, police, and the judicial system. The distinction between ‘legal matters’ and ‘non-legal matters’ is often a grey one in South Africa [24].

Participants also reported their age, education, pregnancy history, relationship length, and cohabitation status. Following previous research in South Africa, we created a measure of socioeconomic status by using principal components analysis to derive a linear index from a series of asset ownership indicators and then categorized participants as belonging to the poorest 40 %, middle 40 %, or wealthiest 20 % on the asset index scale [25–30]. Pregnancy characteristics included parity and gestational age at baseline.

### Statistical analysis

Data on 1480 women (571 HIV-positive, 909 HIV-negative) were analyzed. All statistical analyses were performed in SAS v. 9.3 [31]. First, we calculated estimates of legal knowledge and experiences of legal problems by HIV status. Second, we made statistical comparisons across HIV status for legal knowledge and experiences of legal problems, using t-tests for continuous variables and chi-square tests for dichotomous variables. Lastly, we calculated counts and made descriptive comparisons for assistance seeking for legal issues and barriers to seeking assistance variables due to low sample size. Responses for these two groups of questions were restricted to women who had experienced legal issues, leaving us with a small sample size for these items. For this reason these items are only presented and discussed descriptively.

### Ethical review

The study was approved by the ethical review committees at the University of North Carolina at Chapel Hill and the University of KwaZulu-Natal, Nelson Mandela School of Medicine. Individual written informed consent was obtained from all study participants.

## Results

### Sample characteristics

Table 2 characterizes the total, HIV-positive, and HIV-negative samples. Women varied significantly in all demographic characteristics by HIV status. On average, at baseline women were 25.5 years of age and HIV-positive women were significantly older on average than HIV-negative women (26.4 years vs. 25.0 years, respectively). A greater proportion of HIV-negative women had completed secondary school than HIV-positive women (56.9 % vs. 42.4 %, respectively), and were more likely to be of middle or highest socioeconomic status. About a quarter of all women lived with their partner (25.9 %) and HIV-positive women were more likely to live with their partner than HIV-negative women (28.9 % vs. 24.1 %, respectively). The average duration of women’s current relationships was 4.5 years, with HIV-negative women having longer current relationships than HIV-positive women (4.8 years vs. 3.9 years, respectively).

**Table 2:** Participant Characteristics^a^

		Total (*n* = 1480)	HIV + (*n* = 571)	HIV - (*n* = 909)
Maternal age (y)***		25.5 ± 5.3	26.4 ± 5.1	25.0 ± 5.4
Education level (*ref = less than primary school completed*)***				
	Primary school completed	623 (42.1)	280 (49.0)	343 (37.7)
	Secondary school completed	759 (51.3)	242 (42.4)	517 (56.9)
Socioeconomic status (*ref = lowest SES*)***				
	Middle	573 (38.7)	206 (36.1)	367 (40.4)
	Highest	290 (19.6)	91 (15.9)	199 (21.9)
Lives with partner***		384 (25.9)	165 (28.9)	219 (24.1)
Length of relationship (y)***		4.5 ± 4.1	3.9 ± 3.6	4.8 ± 4.3

^a^Data are expressed as No.(%) or Mean ± SD, unless otherwise indicated

***indicates significant difference by HIV-status where *p* < .05

### Legal knowledge by HIV status

Knowledge of legal rights was high among HIV-positive and -negative women alike, with few significant differences in knowledge by HIV-status. Even where there were significant differences in knowledge, often high proportions of both groups responded to questions correctly. For example, 99 % of HIV-negative vs. 98 % of HIV-positive women were aware of laws requiring fathers to pay child support. Further HIV-negative women were more likely than HIV-positive women to be aware of legal action which women are able to pursue to make the father of their child pay child support (96 % of HIV-negative vs. 94 % of HIV-positive). Lastly, HIV-negative women were more likely than HIV-positive women to know that they can develop a will to designate their possessions to people after their death (83 % vs. 79 %).

Overall, fewer women selected informal sources including family members and village leaders as possible sources of support for legal issues in comparison to formal sources of legal assistance, such as police or legal representation by an attorney. While 88 % of women said that police would help a woman who is being beaten by her partner, only 30 % said that family members could help, and only 26 % said that elders or chiefs could help. 68 % of women said that they could file a maintenance (child support) order to make the father of their child pay child support, while only 6 % said they talk with family to resolve the issue. 53 % of women said that they would file for custody (termed “care” in South Africa) in the event that their partner would want to take their children away without their consent, but only 5 % said that they could talk with relatives to stop their partner from taking the children away. Lastly, to ensure that their possessions go to the right people after their death, the majority of women said that they could develop a will (82 %, with a significantly greater proportion of HIV-negative women responding yes than HIV-positive women), but only 13 % said that they could talk to their relatives to determine who their possessions should be given to (Table 3).

**Table 3:** Legal knowledge by HIV status

Question		Total (*n* = 1480)	HIV+(*n* = 571)	HIV- (*n* = 909)
Do you think it is against the law for a man to beat his partner?		1434 (97)	552 (97)	882 (97)
If a woman is being beaten by her partner, can _______________ help the woman stop the beating?	*Police*	1306 (88)	506 (89)	800 (88)
	*Court*	470 (32)	188 (33)	282 (31)
	*Lawyer*	362 (24)	139 (24)	223 (25)
	*Family*	445 (30)	165 (29)	280 (31)
	*Elder, chief,* etc.	379 (26)	152 (27)	227 (25)
	*No one*	17 (1)	6 (1)	11 (1)
If a man and a woman have a child together, is it against the law if the man does not give the woman any money or support for the child? ***		1453 (98)	554 (97)	899 (99)
Is there anything the woman can do to make the man provide child support? ***		1414 (96)	538 (94)	876 (96)
What can she do?	*Obtain a maintenance order that requires the father of the child to pay*	1010 (68)	382 (67)	628 (69)
	*Talk with family*	82 (6)	34(6)	48 (5)
If a woman’s husband passes away, does the law allow her to receive his death benefits?		1333 (90)	508 (89)	825 (91)
If a man and a woman get divorced, is there a law that protects the woman if she wants to keep the child?		1060 (72)	392 (69)	668 (73)
If the male partner wants to take the children away but the female does not consent, how can the female stop him from taking the children?	*File for custody of her children.*	786 (53)	306 (54)	480 (53)
	*Talk with relatives to stop partner from taking children*	73 (5)	30 (5)	43 (5)
Does the law have a say in what happens to people’s possessions after they pass away?		1078 (73)	401 (70)	677 (74)
How can a person use the legal system to help make sure that their possessions go to right people after they pass away?	Develop a will that specify what should happen to her things when she is gone***	1207 (82)	449 (79)	758 (83)
	Talk with her relatives to determine who should have her things when she is gone	198 (13)	80 (14)	118 (13)

***indicates significant difference in proportions by HIV-status where *p* < .05

Data are expressed as No.(%)

### Legal issues by HIV status

Among reported legal problems, women had most commonly experienced issues obtaining employment (11 %) followed by issues obtaining identification documents (7 %). Very few women had ever felt the need to make a will (.07 %), had experienced divorce or separation (.14 %) or had needed to file for child custody (.75 %). There were few significant differences by HIV-status in experiences of legal problems. The most notable difference was seen in difficulty obtaining employment (15 % of HIV-positive women vs. 9 % of HIV-negative women). Only 6 % of HIV-positive women reported ever needing to file for a maintenance order (child support), but HIV-positive women were more likely to have needed to do so than HIV-negative women, of whom only 4 % had ever had this need. Though few HIV-positive women had experienced health care discrimination, they were more likely to have experienced such discrimination than HIV-negative women (2 % vs. 1 %). Surprisingly, HIV-positive women were less likely to report having experienced discrimination in school than HIV-negative women (0.18 % vs. 2 %) (Table 4).

**Table 4:** Legal issues by HIV status

Legal Problem	Total (*n* = 1480 unless otherwise noted)	HIV + (*n* = 571)	HIV - (*n* = 909)
Housing	56 (4)	19 (3)	37 (4)
Obtaining employment*** (*n* = 1474)	169 (11)	84 (15)	85 (9)
Obtaining residence permit	24 (2)	12 (2)	12 (1)
Obtaining ID document	101 (7)	46 (8)	55 (6)
Making a will	1 (.07)	0 (0)	1 (.11)
Discrimination			
Work	51 (4)	19 (3)	32 (4)
School***	15 (1)	1 (.18)	14 (2)
Health care***	16 (1)	11 (2)	5 (1)
Relationship issues			
Abusive partner (*n* = 1475)	79 (5)	37 (6)	42 (5)
Divorce/separation	2 (.14)	1 (.18)	1 (.11)
Maintenance*** (*n* = 1475)	67 (5)	34 (6)	33 (4)
Custody (*n* = 1475)	11 (.75)	7 (1)	4 (.44)

***indicates significant difference in proportions where *p* < .05

Data are expressed as No.(%)

### Assistance seeking for legal issues

Regardless of HIV status, only a small proportion of women who had experienced legal issues had ever sought help prior to participation in the study. Of the 592 instances of legal issues, assistance had been sought in only 38 % of cases (224). For the most common legal problem, obtaining employment, only 27 % had sought assistance. Other than the single case of making a will, the greatest proportion of assistance seeking was seen for the second most common issue, obtaining an ID document, for which assistance was sought in 61 % of cases. 50 % of women experiencing issues related to obtaining a residence permit, or divorce or separation had sought formal assistance, as had 55 % of women facing custody issues. None of the 16 women who had experienced health care discrimination had sought assistance. For all other legal problems proportions of assistance seeking ranged between 27 % and 49 %.

While few women endorsed family and other sources of informal support as solutions to legal issues in the knowledge portion of the questionnaire, among those who had sought support for legal problems many cases had been resolved through informal means. Over 50 % of cases of legal assistance seeking were addressed through informal or personal sources of assistance including family members and friends. Following informal sources of assistance, the next most common source was the police (32 instances), and the least frequently used source was private legal assistance such as lawyers (4 instances) (Table 5).

**Table 5:** Assistance seeking and source of assistance by legal issue

Problem		Ever had problem^a^n (% of total)	Received legal assistance *n* (% of total with problem)	Where sought assistance *n* (% of total receiving assistance)					
				Private	Nonprofit	Police	Social Work	Government Agencies	Informal/Personal
Housing		56 (4)	12 (21)			1		7	4
Obtaining employment		169 (11)	45 (27)	1				4	30
Obtaining residence permit		24 (2)	12 (50)		1			6	5
Obtaining ID document		101 (7)	62 (61)		1	4	3	2	51
Making a will		1 (.07)	1 (100)						1
Discrimination									
	Work	51 (4)	17 (33)	3	3	2		2	7
	School	15 (1)	8 (53)						8
	Health care	16 (1)	0						
Relationship issues									
	Abusive partner	79 (5)	39 (49)		2	21	1		17
	Divorce/separation	2 (.14)	1 (50)			1			
	Maintenance	67 (5)	21 (31)		7	3	6	3	2
	Custody	11 (.74)	6 (55)		2		2	1	1
TOTAL (instances)		592	224 (38)	4	16	32	12	25	126

^a^4 participants refused to answer all of the questions in this section; 2 participants missing data on all of these questions

### Barriers to seeking assistance for legal issues

As seen above, for most legal issues the majority of women experiencing a given problem did not seek assistance. The most frequently cited reason for not seeking assistance was having no time (78 instances), followed by government bureaucracy (48). Lack of knowledge and the inability to afford legal services were the least commonly cited barriers to seeking assistance (7 instances of each) (Table 6).

**Table 6:** Barriers to seeking assistance for legal issues

Problem	Can’t afford service	Doesn’t know where to go	No time	Didn’t know help was available	Govt. bureaucracy	ID	Knowledge	Personal	Don’t Know/ Not Specified	TOTAL
Housing	3	2	11	4	3	4			29	56
Obtaining employment	2	2	38	6	36		2	1	82	169
Obtaining residence permit		3		5					16	24
Obtaining ID document	1	1	6	2	3	13			75	101
Making a will									1	1
Discrimination										
Work		8	4	7		1	1	1	29	51
School		2		2					11	15
Health care	1	2	3	1			2		7	16
Relationship issues										
Abusive partner		6	8	2			2	7	54	79
Divorce/separation									2	2
Maintenance		5	7	2	6	1		2	44	67
Custody		1	1						9	11
Total (instances)	7	32	78	31	48	19	7	11		

### Discussion

These findings point to a need for assistance for legal issues which is largely un-related to HIV status among this population of childbearing women. Knowledge of rights related to intimate partner violence, divorce, child support, and property appears to be high in this population, and there were few important differences in legal knowledge by HIV - status. On the other hand, there were items on which knowledge was universally low, particularly a low proportion of women consistently said that family and traditional leaders were good sources of support for legal issues. In contrast, though few women sought help for common legal issues, when they did get support they most often sought informal support from family and friends.

Lack of knowledge and the inability to afford legal services were the least commonly cited barriers to seeking assistance. These findings indicate that knowledge may be a comparatively insignificant barrier to seeking legal assistance. Instead, time and convenience may be the most important barriers to assistance seeking. Taken together these findings suggest that although women are aware of formal recourse for key legal issues, they may not seek out formal legal recourse in the event of actually experiencing a legal issue, preferring informal means of resolution.

The most common issues reported, obtaining employment and ID documents, were not strictly legal issues in and of themselves but have important implications for women’s wellbeing and their ability to access government services and exercise their rights. Identification documentation is similar to social security documents in the US and is needed to access numerous government services including housing, education, and some healthcare services, social grants, and the Unemployment Insurance Fund [32]. While not having an identity document is not an absolute preclusion for bringing a case to court in South Africa, the success of the case is greatly hampered by not having such a document. In the early days of ART treatment in South Africa, an ID document was needed to receive treatment, but this requirement was soon removed as it was seen as a barrier to treatment access [33]. As a result many women enrolled in PMTCT programs may not have identification [33], presenting a critical opportunity to help women navigate the process of obtaining an ID document as an essential step toward accessing government services and exercising their rights as new mothers.

Though HIV-positive women have similar levels of knowledge of their legal rights and experience similar levels of legal issues than HIV-negative women in this context, there are implications for the application of these rights which specifically concern HIV-positive women. Access to proper housing has been shown to be protective against negative physical and mental health outcomes among people living with HIV/AIDS [34]. Further, employment has been shown to promote adherence to antiretroviral therapy [35]. HIV-positive women in our sample were more likely to have faced difficulties in obtaining employment than HIV-negative women. The high levels of unemployment in the general population and comparatively higher levels of unemployment among women than among men [36] suggest a need for attention to employment opportunities for all South African women regardless of HIV - status. However, given the higher burden of difficulties finding employment among women living with HIV in our sample, it may be particularly important to provide targeted employment aid to HIV-positive women in this setting. On most legal issues examined here, however, the needs of HIV-positive and - negative women were similar. Access to legal services is a barrier that many women of reproductive age face regardless of HIV-status, and therefore integrating legal and antenatal services would be essential in addressing common legal issues seen in this population.

### Implications

Systemic changes may be warranted to increase women’s access of the legal system. Taking the example of IPV [37], such cases can currently only be addressed in Magistrates' Courts or Family Courts found in urban areas [38]. This poses a major geographic barrier to the 15.5 million South Africans (or 36 % of the population) living in rural areas, the majority of whom are women [39]. Though there are nearly 1500 customary courts operating in South Africa, there are no laws allowing these courts to issue protection orders [40]. Expanding the ability of customary courts to adjudicate routine legal processes such as issuing protection and maintenance orders would greatly increase access to the legal system for the millions of women living in rural areas. The AIDS Council has also suggested promoting the use of pro bono work by private law firms, law clinics, and public interest law centers which could be facilitated through the institution of referral systems at the local level [13]. There is currently no enforcement among South African lawyers in private practice to ensure that they provide a proportion of their time free to the indigent through pro bono work, despite theoretical requirements to do so [41]. Enforcement of these requirements could increase access to the legal system for those most in need.

Another program which could be adapted support to women to facilitate their access and use of legal services is the health navigator model. These navigators have been used in multiple contexts to facilitate health care utilization among underserved populations [42]. These navigators typically come from the same ethnic or linguistic background as the target population and have often personally dealt with the same health issues and thus same health services [42]. A similar model is being implemented in the New York legal system in the United States [43], utilizing paralegal volunteers to help those in need of assistance complete necessary paperwork and accompany litigants to court. A similar model could be implemented in South Africa with the use of volunteers or social workers who have received paralegal training.

Though such proposals hold promise, even if adopted they could take many years to implement. With this in mind, in the short term programs attempting to address the legal needs of this population should focus on reducing the financial and time-related barriers associated with seeking assistance for the most common legal issues found in our sample, namely the issues of unemployment-related matters, lack of housing, obtaining ID documents, IPV, and maintenance orders. In addition to facilitating access to formal assistance, programs should encourage women to better recognize informal sources of support including family members and traditional leaders that they have at their disposal to address employment and housing issues. Further, programs should help women to obtain the personal documentation needed to file legal cases.

### Study limitations

This study is not without limitations. Women were asked to recall their experiences of legal issues retrospectively over an undefined time period, thus it is difficult to know if the experiences are reported encompass all legal issues participants experienced in their adult life. Further, the results in this study cannot represent the experiences of all PLHIV or indeed all women and girls living with HIV in South Africa. Future studies should attempt to document the legal needs of other populations of PLHIV. Results related to barriers to seeking legal services merit further exploration, particularly through qualitative studies to understand the varied and complex difficulties that women face in accessing key legal services. Lastly, the data presented here were collected prior to women knowing their HIV status, thus their experiences of discrimination are not representative of PLHIV whose HIV status could be known by healthcare providers, potential employers, etc.

## Conclusion

Despite these limitations, this study provides important evidence of the legal experiences of a vulnerable population of women. Our findings indicate few differences in legal knowledge and needs between HIV-positive and HIV-negative women in this population, but rather highlight the legal needs common among these women of reproductive age. These results indicate that knowledge may be a comparatively insignificant barrier to seeking assistance for legal issues in this population, and rather time, convenience, and cost may be the most important barriers in this context.

## Abbreviations

ART: Antiretroviral therapy
IPV: Intimate partner violence
PLHIV: People living with HIV/AIDS
PMTCT: Prevention of mother-to-child transmission
SAHAPS: South Africa HIV/AIDS Antenatal Post-test Support Study
